# Therapeutic Effect of Lymph Node Dissection After Neoadjuvant Chemoradiation Therapy Followed by Esophagectomy on Esophageal Squamous Cell Carcinoma Using the Efficacy Index

**DOI:** 10.1111/1759-7714.70057

**Published:** 2025-03-25

**Authors:** Jiyoun Park, Boram Park, Seong Yong Park, Dongryul Oh, Yeong Jeong Jeon, Junghee Lee, Jong Ho Cho, Hong Kwan Kim, Yong Soo Choi, Jae Il Zo, Young Mog Shim

**Affiliations:** ^1^ Department of Thoracic and Cardiovascular Surgery, Samsung Medical Center Sungkyunkwan University School of Medicine Seoul Republic of Korea; ^2^ College of Medicine Inha University Incheon Republic of Korea; ^3^ Department of Radiation Oncology, Samsung Medical Center Sungkyunkwan University School of Medicine Seoul Republic of Korea

**Keywords:** efficacy index, esophageal squamous cell carcinoma, lymph node dissection, neoadjuvant chemoradiation therapy

## Abstract

**Background:**

The effect of lymph node (LN) dissection on the overall survival of patients with esophageal squamous cell carcinoma (ESCC) treated by neoadjuvant chemoradiation therapy (nCRT) followed by esophagectomy has been controversial. This study investigated the patterns of metastatic LNs after nCRT and the benefits of LN dissection using the efficacy index (EI).

**Methods:**

The EI was calculated by multiplying the frequency (%) of metastases to a zone and the 5‐year overall survival rate (%) of patients with metastases to that zone and then dividing by 100. EIs were compared according to the primary lesion location, response to nCRT, and preoperative radiation coverage.

**Results:**

Among 573 patients, the mean age was 62.66 ± 8.10 years, and 533 (93.02%) were male. The mean number of dissected LNs was 37.62 ± 14.76. In all patients, bilateral recurrent laryngeal LNs and paracardial and left gastric LNs showed high EIs compared with other LN stations, and these patterns were maintained regardless of the primary lesion location. The EIs of bilateral recurrent laryngeal LNs and paracardial and left gastric LNs were still high in complete pathologic responders (ypT0) to nCRT and regardless of preoperative radiation coverage.

**Conclusion:**

In ESCC treated with nCRT followed by esophagectomy, the EI of LNs varied between stations. High EIs of bilateral recurrent laryngeal LNs and paracardial and left gastric LNs after nCRT revealed the importance of adequate and complete dissection of these LN stations regardless of the pathologic response to nCRT and the radiation coverage.

## Graphical abstract

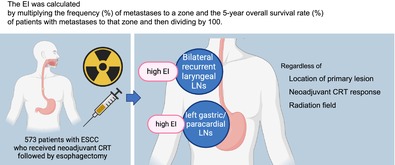

## 1 Introduction

Esophageal cancer ranks as the seventh most common cancer and the sixth leading cause of cancer‐related deaths worldwide. It was responsible for 1 in every 18 cancer‐related deaths in 2020 [1, 2]. Patients with locally advanced esophageal cancer (≥ T2 or node‐positive disease) exhibit poor prognosis, with 5‐year survival rates ranging from 15% to 34% [3]. According to the National Comprehensive Cancer Network (NCCN) guidelines, the standard treatment for locally advanced esophageal cancer is multimodality therapy, including neoadjuvant chemoradiation therapy (nCRT) followed by surgery [4]. Although many studies have confirmed the importance of neoadjuvant therapy with preoperative tumor reduction and eradication of micrometastasis [5], esophagectomy and lymph node (LN) dissection remain the mainstay of treatment, and complete resection is one of the most important prognostic factors in surgically treated esophageal cancer.

The completeness of primary lesion resection can be evaluated by the resection margin (R0 or R1, R2 resection). However, controversies persist regarding the definition and criteria for assessing the quality and completeness of LN dissection in esophageal cancer surgery. Despite being a quality indicator for lymphadenectomy, the number of dissected LNs fails to account for the specific locations of the LN stations dissected. To address this limitation, Sasako et al. [6] proposed the efficacy index (EI), a hypothetical figure that represents the potential effect of dissecting a particular LN region on improving the 5‐year survival rate of an entire cohort. Thus, EI is considered a measure of the effectiveness of LN dissection at a specific station, and the LN station with high EI must be dissected. Several studies have reported the EI in esophageal squamous cell carcinoma (ESCC); however, these studies have analyzed patients who underwent esophagectomy regardless of preoperative therapy (upfront and neoadjuvant therapy altogether), and EI data after nCRT alone is lacking [7, 8, 9, 10]. Because preoperative radiation therapy may eradicate tumor cells in both the primary tumor and metastatic LNs, nCRT may significantly affect the EI. Thus, we hypothesized that the EI after nCRT might be different from the EI after upfront esophagectomy or neoadjuvant chemotherapy followed by esophagectomy and might provide insights into the extent of LN dissection after nCRT. Therefore, this study aimed to investigate the EI in ESCC treated by nCRT followed by esophagectomy.

## 2 Methods

### 2.1 Patients

**FIGURE 1:**
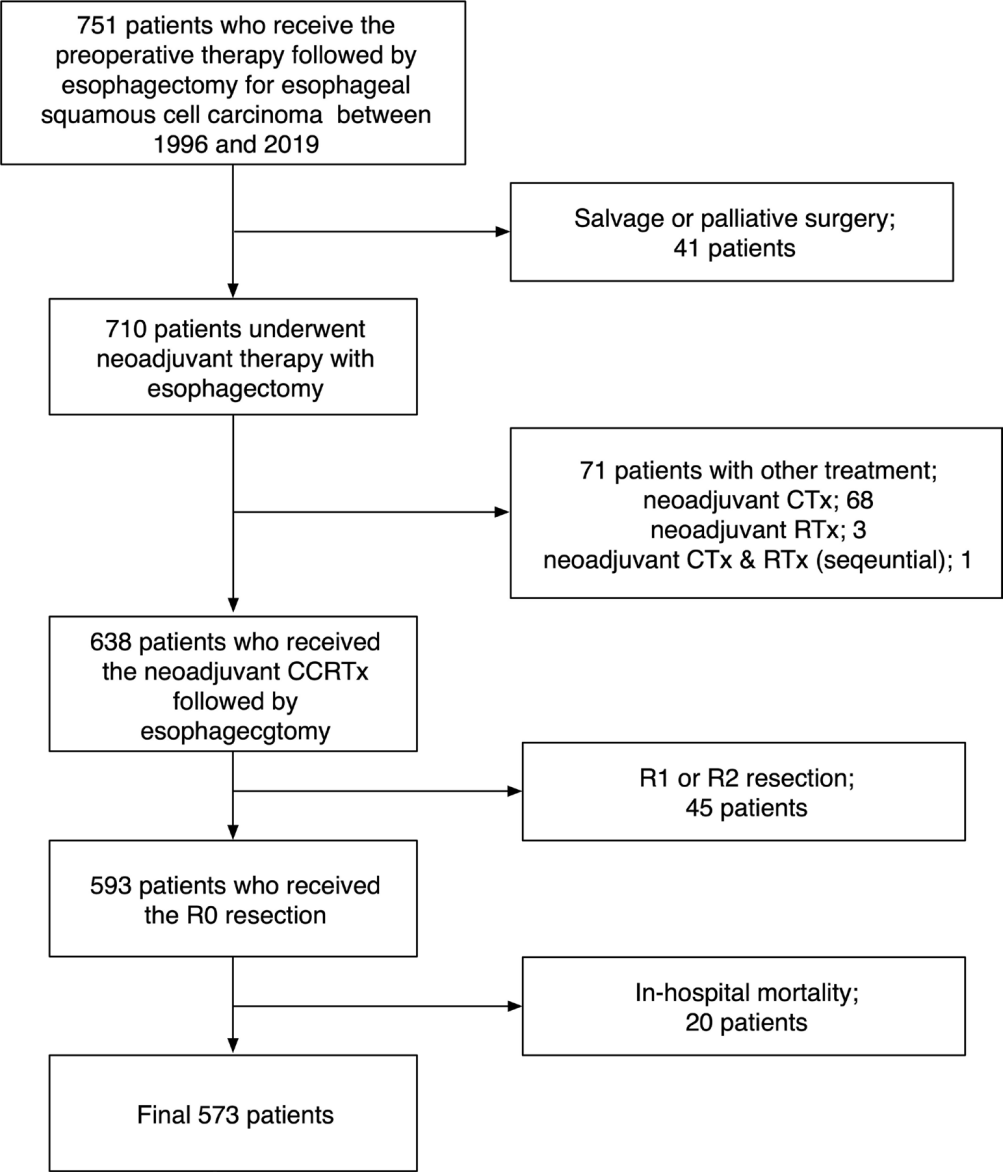
CONSORT diagram of patient selection.

The study included patients with intrathoracic ESCC who received nCRT, followed by esophagectomy between 1996 and 2019, and were identified using the Registry for Thoracic Cancer Surgery, which includes all patients who underwent thoracic surgery at the Samsung Medical Center, Korea, since 1994. The exclusion criteria were as follows: (1) salvage or palliative resection, (2) other types of preoperative therapy such as neoadjuvant chemotherapy, (3) incomplete (R1 or R2) resection, and (4) in‐hospital mortality. Finally, 573 patients were analyzed (Figure 1). The study was approved by the Institutional Review Board (2024‐02‐079), and the need for patient consent was waived because of the retrospective design.

Preoperative evaluation included contrasted chest computed tomography (CT), esophagogastroduodenoscopy, endoscopic ultrasonography, and whole‐body positron emission tomography‐CT (PET‐CT). Two nCRT regimens were used in various periods: Conventional 5‐fluorouracil plus cisplatin (FP) was mainly performed and hypofractionated FP from March 2017 to 2018. Conventional FP consisted of 5‐fluorouracil (5‐FU) 1000 mg/m^2^/day for 4 consecutive days plus cisplatin 60 mg/m^2^/day on Day 1 at 3‐week intervals with 44 Gy daily dose of 2 Gy per fraction (BED10 52.8 Gy). Hypofractionated FP consisted of 5‐FU 1000 mg/m^2^/day for 4 consecutive days plus cisplatin 60 mg/m^2^/day on Day 1 at 3‐week intervals with 43 Gy daily dose of 2.15 Gy per fraction (BED10 52.3 Gy). Involved‐field RT (IFRT) was used: The gross tumor volume (GTV) consisted of the primary tumor and the involved LNs. The clinical target volume (CTV) was defined as the GTV plus 2–3 cm margins in the longitudinal directions, 0.5–1 cm radially for the primary tumor, and 0.5–1 cm margin in all directions for LNs. The planning target volume was applied 0.5 cm from the CTV in all directions. Esophagectomy and reconstruction were performed 4–6 weeks after neoadjuvant therapy. Three‐field LN dissection was performed on patients with upper thoracic esophageal cancer. Neck dissection was also performed when preoperative CT, PET, or PET‐CT indicated cervical LN metastasis in mid‐ to lower thoracic esophageal cancer.

All patients were regularly followed up at 3–4‐month intervals for the first 2 years after surgery and then every 6 months thereafter. Among those who were lost to follow‐up, telephone interviews were conducted to determine the most recent postoperative outcomes. Chest and upper abdomen CTs were performed at every follow‐up appointment, and annual upper endoscopies were performed to rule out disease recurrence. Overall survival (OS) was calculated from the date of surgery to the end of follow‐up (date of death or last contact).

### 2.2 Definition of LN Stations

Regional LNs were defined based on the 11th Japanese Classification of Esophageal Cancer [11]. However, esophagectomy and lymphadenectomy were performed en bloc as an institutional policy; paracardial (1 and 2) and left gastric (7) LNs were dissected together along the stomach. In addition, all paraesophageal LNs were dissected with the esophagus attached, even though the Japanese classification divided them into upper (105), mid‐ (108) and lower paraesophageal (110) LNs. Therefore, paraesophageal (105‐108‐110) and paracardial and left gastric (1‐2‐7) LNs were analyzed as one LN station in this study.

### 2.3 Assessment of the Efficacy of Lymphadenectomy at Each Regional LN Station

The EI was based on the hypothesis that patients with positive nodes in one regional LN who survived 5 years after the resection of positive LNs would not have survived if these LNs were left in situ because dissection of these regional LNs was omitted [6]; therefore, the EI can show the benefit of dissection at a specific LN station. The metastatic rate of each LN station was defined as the frequency of pathologically diagnosed metastases among patients who underwent dissection at each LN station. To assess the efficacy of dissection at each LN station, the EI was calculated by multiplying the metastatic rate (%) at an LN station by the 5‐year OS (%) of the patients who had metastasis at the LN and then dividing by 100 [7, 8]. The 5‐year OS rate of the patients with positive LNs was calculated independently for each LN, irrespective of metastasis to other LNs. The EI was calculated according to the primary lesion location, response to nCRT, and preoperative radiation coverage (in‐field vs. out‐field). Complete pathologic response to nCRT was defined as ypT0 of the primary lesion.

### 2.4 Statistical Analysis

Continuous variables were described as means ± SDs, and categorical variables were described as frequencies with percentages. The median follow‐up time was estimated using the Kaplan–Meier method. The Cox proportional hazards model was used to calculate the hazard ratios (HRs) and 95% confidence intervals (CIs). The two‐proportion *z*‐test was applied to compare the differences between groups for the positive and 5‐year OS rates. All statistical tests were two‐sided, with the significance level set at 0.05, and were performed using STATA 16 (StataCorp LLC, College Station, TX), and R version 4.2.2 (R Project for Statistical Computing).

## 3 Results

### 3.1 Basic Characteristics of the Patients

The basic characteristics of the patients are summarized in Table 1. Of the 573 patients, 167 (29.2%), 234 (40.8%), and 172 (30.0%) had upper, middle, and lower ESCC, respectively. In addition, 279 (48.7%) patients showed complete response to nCRT (ypT0). The mean number of dissected LNs was 37.62 ± 14.76. The median follow‐up period was 56.13 ± 45.90 months.

**TABLE 1:** Basic characteristics of the patients.

Variables	Mean ± SD/frequency (%)
Age	62.66 ± 8.10
Male	533 (93.02%)
Location of lesion	
Upper	167 (29.2%)
Middle	234 (40.8%)
Lower	172 (30.0%)
ypT	
ypT0	279 (48.7%)
ypTis	8 (1.4%)
ypT1	66 (11.5%)
ypT2	114 (20.0%)
ypT3	100 (17.4%)
ypT3	6 (1.0%)
ypN	
ypN0	310 (54.1%)
ypN1	178 (31.1%)
ypN2	65 (11.3%)
ypN3	20 (3.5%)
Mean numbers of dissected LNs	37.62 ± 14.76

Abbreviations: LN, lymph node; SD, standard deviation.

### 3.2 Comparison of the EI According to the Primary Lesion Location

The EIs in all patients and according to the primary lesion location are summarized in Table 2. In all patients, 106recR and 106recL in the mediastinum showed high EI, and paracardial and left gastric LNs in the abdomen also demonstrated high EI. A high EI was observed in the cervical region; however, it must be interpreted cautiously because the number of patients who underwent neck dissection was relatively small, so the positive rate and EI could be overestimated.

**TABLE 2:** Efficacy index in all patients and according to the location of the primary lesion.

Lymph node station	Total 573 patients				Upper ESCC (*n* = 167)				Middle ESCC (*n* = 234)				Lower ESCC (*n* = 172)			
	Dissected patients	Positive rate	5‐year OS	EI	Dissected patients	Positive rate	5‐year OS	EI	Dissected patients	Positive rate	5‐year OS	EI	Dissected patients	Positive rate	5‐year OS	EI
104R (+102R)	193	13.47	40.26%	5.42	108	12.96	54.78%	7.10	65	10.77	42.86%	4.62	20	25.00	0%	0.00
104L (+102L)	199	8.04	35.16%	2.83	113	9.73	42.42%	4.13	66	4.55	33.33%	1.52	20	10.00	0%	0.00
101R	138	11.59	47.54%	5.51	75	12.00	46.30%	5.56	46	8.70	44.34%	3.86	17	17.65	50.88%	8.98
101L	121	17.36	48.20%	8.37	66	21.21	46.43%	9.85	37	18.92	45.01%	8.52	37	0.00	52.61%	0.00
Total cervical region	258	29.46	32.25%	9.50	148	27.70	37.97%	10.52	87	29.89	30.77%	9.20	23	39.13	11.11%	4.35
Paraesophageal	427	6.79	41.18%	2.80	111	7.21	48.84%	3.52	180	7.78	35.29%	2.74	136	5.15	36.00%	1.85
Subcarina (7)	557	6.10	31.91%	1.95	163	4.29	50.00%	2.15	226	7.52	29.17%	2.19	168	5.95	23.08%	1.37
106recR	496	13.91	23.00%	3.20	144	18.75	25.93%	4.86	198	14.14	28.13%	3.98	154	9.09	7.14%	0.65
106recL	497	11.67	21%	2.41	148	11.49	17.65%	2.03	207	13.53	25.00%	3.38	142	9.15	15.38%	1.41
9R	234	1.28	49.40%	0.63	74	1.35	54.17%	0.73	90	2.22	47.87%	1.06	70	0.00	46.55%	0.00
9L	212	4.72	46.97%	2.22	61	3.28	51.55%	1.69	82	2.44	44.44%	1.08	69	8.70	45.31%	3.94
10R	48	0.00	48.07%	0.00	17	0.00	54.47%	0.00	21	0.00	43.06%	0.00	10	0.00	47.90%	0.00
10L	163	2.45	46.78%	1.15	42	0.00	51.29%	0.00	68	2.94	43.52%	1.28	53	3.77	46.07%	1.74
Total mediastinal	572	26.40	26.38%	6.96	167	26.35	29.55%	7.79	234	29.06	29.27%	8.51	171	22.81	17.95%	4.09
Paracardial + left gastric	527	24.86	35.30%	8.77	152	14.47	34.64%	5.01	220	25.91	38.47%	9.97	155	33.55	32.55%	10.92
Common hepatic	495	2.02	29.75%	0.60	149	1.34	45.59%	0.61	195	2.56	43.48%	1.11	151	1.99	43.75%	0.87
Celiac	379	4.75	34.55%	1.64	108	2.78	40.00%	1.11	161	4.35	36.10%	1.57	110	7.27	48.65%	3.54
Total abdominal region	564	25.71	34.47%	8.86	165	15.15	33.94%	5.14	230	26.52	36.35%	9.64	169	34.91	33.67%	11.75

Abbreviations: EI, efficacy index; ESCC, esophageal squamous cell carcinoma; OS, overall survival.

In upper and middle ESCC, bilateral recurrent laryngeal LNs (106recR and 106recL) in the mediastinum and the paracardial and left gastric LNs in the abdomen showed high EIs. The EIs of paracardial and left gastric LNs increased when the primary lesion is located in the lower esophagus. In lower ESCC, the EIs for 104R and 104L were 0% because the 5‐year OS rate in patients with metastasis at 104R and 104L was 0%.

### 3.3 Comparison of the EI According to the Response to nCRT

The EIs according to the response to nCRT are summarized in Table 3. In 279 patients with ypT0, 106recR and 106recL showed high EI (3.83 and 2.45, respectively). The EIs of 106recR and 106recL were still high in the ypT+ group (2.63 and 2.35, respectively). The positive rates of each LN station were high in the ypT+ group, whereas the 5‐year OS was higher in the ypT0 group.

**TABLE 3:** Efficacy index according to the response to neoadjuvant chemoradiation therapy.

Lymph node station	ypT0 patients (*n* = 279)					ypT+ patients (*n* = 294)					*p*	
	Dissected group	Positive group	Positive rate	5‐year OS	EI	Dissected group	Positive group	Positive rate	5‐year OS	EI	Positive rate	5‐year OS
104R (+102R)	91	11	12.09%	45.45%	5.49	102	15	14.71%	37.28%	5.48	0.595	0.680
104L (+102L)	90	3	3.33%	33.30%	1.11	109	13	11.93%	35.90%	4.28	0.026	0.932
101R	71	4	5.63%	62.85%	3.54	67	12	17.91%	34.43%	6.17	0.024	< 0.001
101L	58	4	6.90%	63.71%	4.39	63	17	26.98%	35.34%	9.54	0.004	< 0.001
Total cervical region	119	21	17.65%	57.14%	10.08	139	55	39.57%	22.67%	8.97	< 0.001	0.005
Paraesophageal	207	5	2.42%	69.05%	1.67	220	24	10.91%	21.67%	2.36	< 0.001	< 0.001
Subcarina (7)	272	7	2.57%	66.67%	1.72	285	27	9.47%	20.00%	1.89	0.001	0.002
106recR	235	20	8.51%	45.00%	3.83	261	49	18.77%	13.99%	2.63	0.001	0.011
106recL	242	14	5.79%	42.31%	2.45	255	44	17.25%	13.64%	2.35	< 0.001	0.045
9R	108	1	0.93%	69.44%	0.64	126	2	1.59%	29.57%	0.47	0.654	< 0.001
9L	102	4	3.92%	68.14%	2.67	110	6	5.45%	28.29%	1.54	0.599	< 0.001
10R	22	0	0.00%	65.05%	0.00	26	0	0.00%	32.76%	0.00		< 0.001
10L	77	0	0.00%	63.64%	0.00	86	4	4.65%	30.96%	1.44	0.055	< 0.001
Total mediastinal	279	44	15.77%	52.22%	8.24	293	107	36.52%	15.78%	5.76	< 0.001	< 0.001
Paracardial + left gastric	255	50	19.61%	62.56%	12.27	272	81	29.78%	18.35%	5.46	0.007	< 0.001
Common hepatic	240	2	0.83%	68.18%	0.57	255	8	3.14%	22.17%	0.70	0.069	< 0.001
Celiac	188	0	0.00%	62.15%	0.00	191	0	0.00%	28.25%	0.00		< 0.001
Total abdominal region	273	56	20.51%	58.55%	12.01	291	89	30.58%	20.43%	6.25	0.006	< 0.001

Abbreviations: EI, efficacy index; OS, overall survival.

### 3.4 Comparison of the EI According to the Radiation Field

The EIs according to the preoperative radiation coverage were also investigated (Table 4). In addition, 214 (37.3%), 394 (68.7%), and 189 (33.0%) patients received radiation therapy at the neck, upper mediastinum, and abdominal region, respectively. In the neck region, the EIs of patients who received radiation therapy (in‐field) showed high EI in bilateral 101 and 104. Patients with metastasis at bilateral 104 without preoperative radiation showed a 5‐year OS rate of 0%; therefore, EIs were also 0. In the upper mediastinal and abdominal regions, the EIs of bilateral 106rec and paracardial and left gastric LNs were high regardless of the radiation coverage.

**TABLE 4:** Efficacy index according to the radiation field.

Lymph node station	In‐field					Out‐field					*p*	
	Dissected group	Positive group	Positive rate	5‐year OS	EI	Dissected group	Positive group	Positive rate	5‐year OS	EI	Positive rate	5‐year OS
Neck region												
104R (+102R)	124	22	17.74%	47.91%	8.50	55	2	3.64%	0.00%	0.00	0.011	
104L (+102L)	126	12	9.52%	38.89%	3.70	57	1	1.75%	0.00%	0.00	0.058	
101R	87	8	9.20%	46.45%	4.27	38	4	10.53%	50.54%	5.32	0.816	0.497
101L	70	12	17.14%	47.06%	8.07	39	4	10.26%	51.15%	5.25	0.330	0.500
Upper mediastinum												
106recR	178	32	17.98%	26.63%	4.79	280	32	11.43%	16.67%	1.91	0.049	0.422
106recL	186	22	11.83%	20.00%	2.37	268	29	10.82%	36.36%	3.93	0.738	0.301
Abdominal region												
Paracardial + left gastric	197	42	21.32%	41.96%	8.95	283	73	25.80%	34.46%	8.89	0.258	0.359

Abbreviations: EI, efficacy index; OS, overall survival.

### 3.5 Risk Factors for the OS

Multivariable analysis was performed to find the risk factors for OS (Table 5). In all patients and subset analysis of the primary lesion location, pathologic states such as ypT and ypN were associated with OS, whereas metastasis to a specific region was not related to OS.

**TABLE 5:** Multivariable analysis for overall survival in all patients and according to the primary lesion location.

	All patients		Upper‐ESCC group		Mid‐ESCC group		Lower‐ESCC group	
	HR (95% CI)	*p*	HR (95% CI)	*p*	HR (95% CI)	*p*	HR (95% CI)	*p*
Age	1.011 (0.997–1.025)	0.136	1.002 (0.974–1.030)	0.915	1.021 (0.998–1.045)	0.079	1.009 (0.981–1.037)	0.547
Sex (male vs. female)	1.403 (0.851–2.312)	0.184	1.964 (0.696–5.542)	0.202	1.700 (0.717–4.029)	0.228	0.872 (0.389–1.953)	0.739
ypT (vs. ypT0)								
ypTis	2.886 (1.335–6.238)	0.007	8.117 (1.898–34.711)	0.005	4.433 (1.342–14.640)	0.015	1.358 (0.311–5.924)	0.684
ypT1	1.583 (1.116–2.246)	0.010	1.830 (0.995–3.369)	0.052	1.589 (0.935–2.699)	0.087	1.064 (0.481–2.351)	0.879
ypT2	1.526 (1.141–2.041)	0.004	1.531 (0.867–2.704)	0.142	1.071 (0.672–1.707)	0.772	2.318 (1.353–3.970)	0.002
ypT3	2.063 (1.526–2.788)	< 0.001	2.036 (1.177–3.522)	0.011	1.874 (1.147–3.061)	0.012	2.199 (1.207–4.006)	0.010
ypT4	6.337 (2.752–14.592)	< 0.001	8.849 (1.828–42.844)	0.007	14.059 (1.681–117.568)	0.015	5.907 (1.723–20.246)	0.005
ypN (vs. ypN0)								
ypN1	1.401 (0.885–2.218)	0.150	1.006 (0.417–2.429)	0.989	1.162 (0.585–2.308)	0.669	1.955 (0.769–4.973)	0.159
ypN2	2.542 (1.251–5.168)	0.010	0.906 (0.224–3.664)	0.890	2.311 (0.791–6.753)	0.126	7.256 (1.807–29.126)	0.005
ypN3	2.505 (1.119–5.606)	0.025	0.889 (0.198–3.988)	0.878	3.217 (0.866–11.949)	0.081	5.658 (1.171–27.336)	0.031
Metastasis to cervical region	0.802 (0.540–1.192)	0.275	1.050 (0.527–2.092)	0.889	0.942 (0.521–1.702)	0.843	0.878 (0.316–2.441)	0.803
Metastasis to mediastinal region	1.331 (0.888–1.995)	0.166	1.952 (0.865–4.405)	0.107	1.333 (0.734–2.422)	0.345	1.255 (0.534–2.949)	0.603
Metastasis to abdominal region	1.046 (0.712–1.537)	0.819	1.362 (0.622–2.983)	0.440	1.054 (0.578–1.922)	0.864	0.854 (0.389–1.877)	0.695

Abbreviations: CI, confidence interval; ESCC, esophageal squamous cell carcinoma; HR, hazard ratio.

## 4 Discussion

This study showed that the EIs of bilateral recurrent laryngeal nerve LNs and paracardial and left gastric LNs were still high after nCRT followed by surgery. The response to nCRT and radiation therapy coverage appeared not to alter the EI patterns. In addition, the metastasis to specific regions was not related to poor survival after adjusting for the ypT and ypN.

The optimum extent of lymphadenectomy for esophageal cancer has been debated, and no consensus has been established on the definition of optimal lymphadenectomy in esophagectomy. The current NCCN guideline suggests that a minimum of 15 LNs must be dissected during esophagectomy for accurate staging and optimal survival [4]. Several studies have reported that the number of dissected LNs is related to survival after neoadjuvant therapy followed by esophagectomy [12]. Although many studies have reported the number of dissected LNs as a quality parameter for LN dissection [13], this number cannot reflect the location of the dissected LN station. If LN dissection focuses exclusively on regions where metastasis is less common, neglecting the sites where it frequently occurs, the total number of LNs removed may still meet the criteria that are based on the number of LNs.

Because the number of dissected LNs could not be a sufficient indicator for optimal lymphadenectomy, the EI has been applied in gastric and esophageal cancer. As mentioned in the methods section, therefore, the EI can show the benefit of dissection at a specific LN station, under the hypothesis that patients with positive nodes in one regional LN who survived 5 years after the resection of positive LNs would not have survived if these LNs were left in situ because dissection of these regional LNs was omitted [6]. A handful of studies have reported the EI in ESCC and the higher EI in bilateral recurrent laryngeal LNs (106recR and 106recL) and paracardial LNs (1 and 2) than in other LN stations [7, 8, 9, 10]. Because the EI can be altered by dissection patterns, treatment modalities, the definition of LN station, and patients' characteristics [8], the direct comparison of the absolute EI within the published studies may be inappropriate, so it must be interpreted within each study. However, previous studies have reported similar patterns that bilateral recurrent laryngeal nerve LNs and paracardial LNs showed high EI, and these results have been used as evidence to support the importance of complete dissection of these LN stations.

Theoretically, the EI can be changed according to the preoperative therapy. Neoadjuvant therapy has been reported to not only decrease the frequency of LN metastases but also change the distribution of LN metastases [14, 15, 16], and these effects could change the EI compared with the EI of patients who received upfront surgery. However, previous studies have analyzed upfront esophagectomy and neoadjuvant therapy followed by esophagectomy [7, 8, 9, 10]; therefore, these studies could not verify the potential changes of the EI according to the effect of preoperative therapy. The relationship between preoperative therapy and the EI could be an important issue because it is related to the extent of lymphadenectomy. Some investigators have proposed that the extent of lymphadenectomy can be minimized after neoadjuvant therapy for esophageal cancer [14, 17, 18]. Talsma et al. [14] reported that the number of resected LNs correlated with survival after surgery alone but not after nCRT and concluded that the benefit of maximal LN dissection after preoperative CRT is questionable by analyzing the results of the CROSS study. Conversely, other studies have emphasized the clinical importance of extensive lymphadenectomy on patient survival even after neoadjuvant therapy [19, 20, 21, 22]. Therefore, understanding EI changes according to neoadjuvant therapy is important to address controversies on whether less‐extensive lymphadenectomy can be appropriate after neoadjuvant therapy.

Miyata et al. [23] examined the EI of LN dissection for each LN station in patients with ESCC who received neoadjuvant chemotherapy. Even after neoadjuvant chemotherapy, bilateral recurrent laryngeal LNs and paracardial LNs still showed high EI, irrespective of the tumor location, similar to patients without neoadjuvant therapy. In addition, the EI for each LN station did not vary according to the response to neoadjuvant therapy. They concluded that the EI of each LN was not affected by neoadjuvant chemotherapy; therefore, complete LN dissection is important after neoadjuvant chemotherapy. In addition, they suggested that the EI patterns after nCRT might be different to the neoadjuvant chemotherapy or upfront esophagectomy because nCRT shows better locoregional controls than neoadjuvant chemotherapy in general [23]. In this study that analyzed patients who received nCRT only, bilateral recurrent laryngeal nerve nodes (106recR and 106recL) and paracardial LNs (1 and 2) still showed high EI, and the EI was not changed according to the pathologic response or radiation coverage. This finding showed that even after nCRT, the EI patterns are similar to upfront surgery or neoadjuvant chemotherapy followed by surgery, and the concept of “less‐extensive lymphadenectomy after nCRT” is not still valid.

Interestingly, in this study, we calculated the EI according to the coverage of preoperative radiation therapy. When evaluating the locoregional control of nCRT, considering the field of radiation is critical. Radiation may include elective LN irradiation (ENI) depending on the location of the primary tumor or may be guided by delineating appropriate margins from the primary tumor and involved LNs, a practice referred to as IFRT, by the references and policies of the radiation oncologist. Hanami et al. analyzed 184 patients with ESCC who were treated with nCRT followed by surgery, and ENI was used [24]. In this analysis, although approximately 50% of patients who were clinically diagnosed with LN metastasis before treatment were downstaged by nCRT, LN metastases were extensive in cervical, mediastinal, and abdominal areas, even within the radiation field. They concluded that systematic and adequate lymphadenectomy is essential after nCRT with ENI and esophagectomy, but they did not calculate EI. In our institution, we applied IFRT, and extensive lymphadenectomy was performed as an institutional policy. Our results showed that the EI patterns were not different according to the preoperative radiation coverage. In upper esophageal cancer, although the upper mediastinum was routinely covered by preoperative radiation therapy, the EIs of 106recR and 106recL were still high. These findings showed that even in the LN station that received preoperative radiation with IFRT, extensive and adequate lymphadenectomy is mandatory.

The definition of the N factor in ESCC, between the number of LN metastases and the location of metastatic LNs, is still controversial. If the survival rate varies depending on the presence of metastases at specific regions or in specific LN stations, even if these are defined as the same regional LNs, these LNs could be defined as a high N stage or distant metastasis (M1) rather than regional LNs. Regarding this problem, Kanemura et al. [9] analyzed both the EI and recurrence patterns of each LN station and suggested that the LN station with low EI and high recurrence rate must be classified as M1 instead of regional LNs. Miyata et al. [25] reported that metastasis to a specific LN station such as the middle mediastinal region and celiac LNs is related to the OS in patients with neoadjuvant chemotherapy followed by surgery, whereas our results showed that metastasis to specific regions was not related to survival. The definition of the N stage and regional LNs must be studied with large data in the future.

This study had some limitations. First, not all LN stations were dissected in all patients. Specifically, because neck dissection was performed in cases in which clinically metastatic LNs are suspicious, the positivity rate can be overestimated; therefore, the EI of the neck nodes can also be overestimated. Second, the definition of the LN station in this study is slightly different from the Japanese classification [11], particularly regarding paraesophageal and left gastric, and paracardial LNs. Third, the response to nCRT was classified only by the pathologic response in the primary tumors; accurate assessment of the response in metastatic LNs appears challenging. Finally, changes in EIs after nCRT must be compared with EIs after upfront surgery; however, it is not possible because neoadjuvant therapy followed by surgery is the standard therapy in locally advanced ESCC. Despite these limitations, this is the first study that analyzed patients who received nCRT followed by esophagectomy only and compared the EIs according to the response to nCRT and radiation field.

In conclusion, this study showed that the EIs of bilateral recurrent laryngeal LNs and paracardial and left gastric LNs were high after nCRT followed by surgery. The response to nCRT and radiation field did not alter the EI patterns, high EIs of bilateral recurrent laryngeal LNs and paracardial and left gastric LNs after nCRT revealed the importance of adequate and complete dissection of these LN stations regardless of the pathologic response to nCRT and the radiation coverage. It also indicated that the concept of “less‐extensive lymphadenectomy after nCRT” might not be valid.

## Author Contributions

**Seong Yong Park:** concept and design. **Boram Park, Seong Yong Park, Dongryul Oh:** acquisition, analysis, or interpretation of data. **Jiyoun Park, Seong Yong Park, Boram Park, Dongryul Oh:** drafting of the manuscript. **Jiyoun Park, Boram Park, Seong Yong Park, Dongryul Oh, Yeong Jeong Jeon, Junghee Lee, Jong Ho Cho, Hong Kwan Kim, Yong Soo Choi, Jae Il Zo, Young Mog Shim:** critical revision of the manuscript for important intellectual content. **Boram Park:** statistical analysis. **Seong Yong Park, Hong Kwan Kim:** obtained funding. **Seong Yong Park:** administrative, technical, or material support.

## Conflicts of Interest

The authors declare no conflicts of interest.

## Data Availability

The data used in this study are available upon reasonable request from the corresponding author. However, access to the data is subject to the completion of the data transfer agreement process required by the affiliated institution.
